# The Orthoplastic Approach: Principles Integrating Orthopedic and Plastic Surgery in Trauma Care

**DOI:** 10.7759/cureus.112215

**Published:** 2026-07-07

**Authors:** Diego-Gerardo Luna-Pérez, Stephanie-Johanna Chon-Pineda, Alfonso Sandoval, Jorge-Alejandro Moreno-de la Paz, Diego González-Sanvicente, Javier-Emilio Santos-Salazar, Jeffrey Barragan-Ortega, María-Fernanda Aguirre-Quintero, Aarón Gonzalez Espinosa, José-Emiliano González-Flores

**Affiliations:** 1 School of Medicine and Health Sciences, Tecnológico de Monterrey - Campus Ciudad de Mexico, Mexico City, MEX; 2 Medical Oncology, Instituto Nacional de Ciencias Médicas y Nutrición Salvador Zubirán, Mexico City, MEX; 3 Plastic and Reconstructive Surgery, Hospital General de México “Dr. Eduardo Liceaga”, Mexico City, MEX; 4 Medical Research and Academic Mentorship, ShareTheMed Research Group, Mexico City, MEX; 5 Faculty of Public Health, Universidad Anáhuac México, Mexico City, MEX

**Keywords:** complex extremity trauma, fix-and-flap strategy, functional recovery, limb salvage, lower extremity reconstruction, multidisciplinary care, open fracture management, orthoplastic surgery, reconstructive plastic surgery, soft tissue reconstruction

## Abstract

Complex extremity trauma presents a major reconstructive challenge because it simultaneously involves skeletal injury, soft-tissue loss, vascular compromise, contamination, and functional impairment. The orthoplastic approach has emerged as an integrated model that promotes early collaboration between orthopedic trauma and reconstructive plastic surgery teams to optimize limb salvage and recovery.

This structured narrative review examined the principles, reconstructive strategies, organizational models, and clinical implications of orthoplastic management through a structured search of PubMed, Embase, and Web of Science. The reviewed literature consistently demonstrated that early multidisciplinary reconstruction, particularly protocol-driven orthoplastic pathways and coordinated fix-and-flap strategies, was associated with lower infection rates, improved reconstructive efficiency, higher limb-salvage rates, and better functional recovery. Conversely, delayed or fragmented management was consistently linked to increased complications, prolonged reconstruction, and less favorable outcomes. Beyond surgical technique, successful reconstruction depended on coordinated multidisciplinary planning and systems-based organization. Overall, the orthoplastic approach represents a paradigm shift in contemporary trauma care, emphasizing integrated, time-sensitive reconstruction to achieve durable limb preservation and functional restoration.

## Introduction and background

Complex extremity trauma represents one of the most demanding challenges in contemporary surgical care because it simultaneously involves osseous injury, soft-tissue loss, vascular compromise, neurological damage, and a high risk of contamination and infection. Beyond the immediate threat to limb viability, these injuries are associated with repeated surgical interventions, prolonged hospitalization, functional disability, and significant deterioration in quality of life [1,2].

Historically, management has relied on a sequential model in which skeletal stabilization and soft tissue reconstruction are approached as separate processes. Although rooted in the traditional evolution of surgical subspecialties, this fragmented strategy may result in delayed definitive coverage, disjointed decision-making, and prolonged exposure of critical structures to infection, necrosis, and reconstructive failure [1,3].

The orthoplastic approach emerged to provide a more integrated framework for the management of complex extremity trauma. By promoting early and coordinated collaboration between orthopedic trauma and reconstructive plastic surgery teams, this model conceptualizes the injured extremity as a single biological and functional unit. Rather than addressing bone, vascular, and soft-tissue integrity independently, orthoplastic management emphasizes their interdependence within a unified reconstructive strategy. Evidence from specialized orthoplastic and high-volume trauma centers has associated this model with improved limb salvage rates, lower infectious complications, fewer unplanned procedures, and more efficient reconstructive timelines [1-4].

Despite its growing relevance, adoption of the orthoplastic model remains inconsistent across institutions and healthcare systems due to differences in multidisciplinary team availability, reconstructive expertise, microsurgical infrastructure, referral pathways, institutional protocols, and resource allocation. These factors continue to determine the feasibility of delivering coordinated extremity reconstruction, particularly in resource-variable settings [2,4].

Furthermore, much of the existing orthoplastic literature has focused predominantly on technical procedures and isolated surgical outcomes, whereas the broader integration of orthopedic, reconstructive, biological, and organizational principles remains comparatively underexplored [1-4]. As a result, the orthoplastic approach is often reduced to an interdisciplinary collaboration rather than recognized as a time-sensitive, outcome-driven model of integrated trauma care.

Accordingly, the present narrative review aims to examine the fundamental principles of the orthoplastic approach in complex extremity trauma, with particular attention to its clinical, biological, and organizational foundations. In addition, this review analyzes its relevance for surgical decision-making, trauma system organization, functional outcomes, and the challenges associated with implementing coordinated orthoplastic care across diverse healthcare environments.

## Review

Methods

Study Design

This article was designed as a structured narrative review. A transparent and structured approach was used to identify, select, and synthesize relevant literature. This process was not intended to constitute a formal systematic review, but rather to provide methodological clarity and consistency for a narrative synthesis.

Eligibility criteria were established a priori to guide literature selection. An internal methodological framework was developed by the research team before the article review process. No formal external registration was performed. Given the significant heterogeneity across study designs, injury patterns, interventions, institutional models, and reported outcomes, a quantitative meta-analysis was not performed. Instead, findings were synthesized narratively to identify recurring clinical, reconstructive, and organizational themes.

Search Strategy

A structured literature search was conducted in PubMed, Embase, and Web of Science, covering publications from 1985 to 2025. Search terms combined Medical Subject Headings and free-text keywords related to orthoplastic reconstruction, extremity trauma, open fractures, limb salvage, soft tissue coverage, fixation, and reconstructive timing. Boolean operators “AND” and “OR” were used to combine concepts.

The following search strategy was applied and adapted to each database when required: (orthoplastic OR “ortho-plastic” OR “combined orthopedic and plastic surgery” OR “multidisciplinary reconstruction”) AND (“extremity trauma” OR “limb trauma” OR “open fracture” OR “Gustilo” OR “lower limb trauma” OR “upper limb trauma”) AND (“soft tissue coverage” OR flap OR reconstruction OR “fix and flap” OR “limb salvage”) AND (infection OR amputation OR “functional outcome” OR “clinical outcome” OR “reoperation” OR “hospital stay”).

The search was limited to the selected databases (PubMed, Embase, and Web of Science). Grey literature, conference abstracts, preprints, and unpublished reports were not included. Only articles with full text available were considered. These restrictions may have limited the comprehensiveness of the search but were consistent with the narrative purpose of the review and the aim of synthesizing peer-reviewed clinical literature.

Eligibility Criteria

Eligible article types included observational cohorts, comparative studies, clinical case series, and selected guidelines or review articles used for contextual framing. Review articles and clinical practice guidelines were not treated as primary outcome-generating evidence.

The target population included adult and pediatric patients with complex extremity trauma, particularly open fractures, including high-grade Gustilo-Anderson injuries, accompanied by soft-tissue defects, exposed bone, vascular compromise, tendon exposure, or other complex reconstructive requirements. Timing from injury to definitive fixation or soft-tissue coverage was considered a key clinical variable when reported. Articles were required to address an orthoplastic or multidisciplinary reconstructive approach involving orthopedic trauma and plastic reconstructive surgery teams. Relevant outcomes included infection, limb salvage, amputation, reoperation, timing of definitive coverage, length of hospital stay, complications, functional outcomes, or patient-centered recovery measures. Articles published in English or Spanish between 1985 and 2025 were considered.

Exclusion Criteria

Editorials, letters to the editor, expert opinions without substantive clinical data, and articles focused exclusively on isolated surgical techniques were excluded. Articles centered only on flap design, technical modifications, biomechanical models, computational simulations, animal studies, or non-traumatic reconstruction were not considered eligible. Publications addressing oncologic, congenital, aesthetic, or other non-traumatic reconstructive indications were also excluded. Articles were excluded if they did not provide clinically relevant information related to trauma reconstruction, infection, amputation, limb salvage, reconstructive timing, reintervention, or function.

Literature Selection

All records identified through the database search were exported to a structured spreadsheet in Google Sheets (Google, Inc., Mountain View, CA, USA) for management. Duplicate records were identified and removed manually by two reviewers before title and abstract review. A total of 198 records were identified; 79 were removed as duplicates, leaving 119 unique records for title and abstract screening.

**Figure 1:**
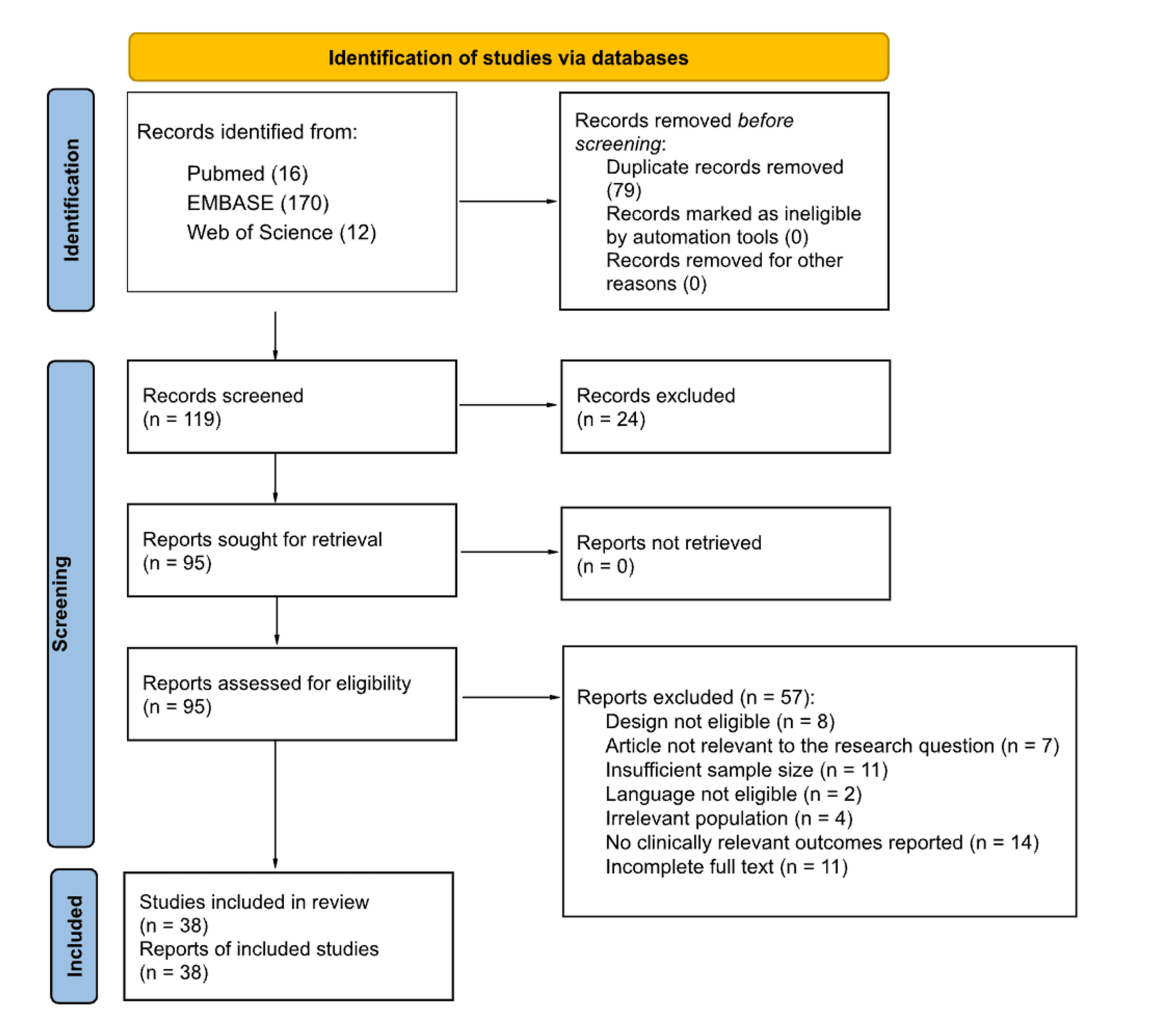
PRISMA-inspired flow diagram of literature identification, selection, and inclusion process for the structured narrative review Flow diagram illustrating the identification, screening, eligibility assessment, and inclusion of studies evaluating orthoplastic management in complex extremity trauma. Articles were identified through structured database searches, followed by duplicate removal, title and abstract review, full-text assessment, and final inclusion in the narrative synthesis according to predefined eligibility criteria.

After initial review, 95 articles were selected for full-text assessment. Full-text articles were evaluated according to the predefined eligibility criteria. Ultimately, 38 articles were included in the narrative synthesis in Figure 1. Article selection was performed by two reviewers, and disagreements were resolved through consensus. 

Data Extraction

Data were extracted using a standardized spreadsheet developed by the research team. Extracted variables included study characteristics, country, publication year, recruitment period, study design, sample size, population characteristics, injury type, anatomical location, trauma severity, and follow-up duration.

Orthoplastic-specific variables included early multidisciplinary collaboration, reconstructive timing, use of fix-and-flap strategies, institutional care model, definitive soft tissue coverage, and coordination between orthopedic and plastic surgery teams. Clinical variables included infection, limb salvage, amputation, reoperation, number of surgical procedures, length of hospital stay, complications, functional outcomes, and patient-centered measures when available. Methodological variables, including comparator group, study design, sample size, follow-up duration, and critical appraisal notes, were recorded to contextualize the strength of evidence.

Quality Assessment

Given the narrative design of this review and the heterogeneity of the available literature, no formal risk-of-bias assessment was performed. Instead, key methodological characteristics - including study design, the presence or absence of a comparator group, sample size, follow-up duration, and outcome reporting - were extracted and integrated into the synthesis according to their relevance to the predefined thematic domains.

This approach allowed the evidence to be interpreted within its methodological context without applying a quantitative quality score. Articles with stronger study designs, clearer outcome definitions, comparative groups, and longer follow-up were given greater interpretive weight during the narrative synthesis.

Data Synthesis

Statistical pooling was not performed because of heterogeneity in study design, patient populations, injury characteristics, institutional models, interventions, and outcome reporting. A narrative synthesis was conducted.

Included articles were organized into thematic domains: core orthoplastic principles, reconstructive timing, skeletal stabilization and soft-tissue coverage, clinical outcomes, institutional models of care, application in resource-variable settings, limitations of the available evidence, and future research directions. These domains were selected because they reflect the central dimensions identified in the included literature: orthoplastic principles, reconstructive timing, skeletal stabilization, soft-tissue coverage, clinical outcomes, organizational models, and evidence gaps in complex extremity trauma. Findings were synthesized qualitatively to identify recurring concepts, clinically relevant patterns, and gaps in the literature.

Results

Of the 38 included articles, primary outcome-generating evidence was derived from observational and interventional studies, while reviews and guidelines were used to contextualize key concepts and recommendations addressing orthoplastic principles, reconstructive timing, skeletal stabilization, soft-tissue coverage, clinical outcomes, organizational models, and evidence gaps in complex extremity trauma [5-42]. The principal models of orthoplastic care and their reported clinical outcomes across the included studies are summarized in Table 1. The timing of reconstruction, fixation, and definitive soft-tissue coverage varied across the included studies and represented one of the most consistently evaluated variables influencing infection, limb salvage, and reconstructive outcomes. The principal reconstructive timing strategies and their reported clinical implications are summarized in Table 2.​​​​​

**Table 1:** Models of orthoplastic care and reported outcomes in complex extremity trauma Abbreviation: BOAST4, British Orthopaedic Association Standards for Trauma and Orthopaedics.

Model of orthoplastic care	Representative studies	Organizational characteristics described in the literature	Timing characteristics reported	Main outcomes reported	Key observations reported by the authors
Protocol-driven orthoplastic care	Ylitalo et al. [9]; Lacey et al. [31]; Wang et al. [15]	Formal orthoplastic collaboration, multidisciplinary pathways, institutional protocols, coordinated orthopedic and plastic surgery management	Early coordinated coverage emphasized; adherence to BOAST4 or institutional timing targets evaluated	Lower complication rates, low deep infection rates, and high limb salvage rates	Authors reported that institutional coordination and formal orthoplastic pathways improved consistency of care and reconstructive outcomes
Early “fix-and-flap” reconstruction	Asano et al. [21]; Wang et al. [15]; Lacey et al. [31]	Early fixation combined with vascularized soft-tissue coverage	Coverage commonly targeted within the first 72 hours	Reduced fracture-related infection, lower reoperation burden, and favorable bone union outcomes	Early coordinated reconstruction was associated with improved infection control and reconstructive efficiency
Staged orthoplastic reconstruction	Nishida et al. [20]; Müller et al. [11]; Jagosz et al. [12]	Sequential debridement, delayed flap coverage, staged fixation, or reconstruction strategies	Delayed reconstruction used when immediate coverage is not feasible	Bone union achieved in selected cohorts; infection-related complications remained clinically relevant	Authors emphasized the role of staged reconstruction in contaminated wounds, fracture-related infection, and complex defects
Fragmented or delayed interdisciplinary care	Higgin et al. [34]; Farhat et al. [38]	Delayed reconstructive involvement, non-standardized pathways, and limited multidisciplinary integration	Delayed coverage or prolonged reconstruction intervals reported	Higher complication burden, prolonged hospitalization, and increased infection rates	Delayed coordination was associated with more complex reconstructive courses and greater morbidity
Resource-limited or humanitarian orthoplastic care	Farhat et al. [38]; Koenig et al. [39]	Orthoplastic collaboration adapted to conflict or resource-variable settings	Timing dependent on logistics and local surgical capacity	Limb salvage remained feasible despite high injury severity	Studies highlighted the importance of coordinated multidisciplinary management even in austere environments

**Table 2:** Reconstructive timing strategies and reported clinical implications in complex extremity trauma

Reconstructive timing strategy	Representative studies	Timing definition reported	Clinical context reported	Main outcomes reported	Key observations reported by authors
Early definitive coverage (“fix-and-flap”)	Lacey et al. [31]; Ylitalo et al. [9]; Wang et al. [15]; Asano et al. [21]	Coverage targeted within the first 72 hours or within the early reconstructive window	Gustilo-Anderson IIIB/IIIC open fractures managed through coordinated orthoplastic pathways	Lower deep infection rates, high limb salvage rates, and fewer reconstructive procedures	Authors emphasized the importance of coordinated early fixation and vascularized soft-tissue coverage
Early reconstruction after limited debridement intervals	Cao et al. [30]	Reconstruction performed after fewer debridement procedures and shorter reconstruction intervals	Severe lower-extremity open fractures	Improved clinical outcomes and shorter reconstructive course compared with delayed strategies	Earlier reconstruction was associated with more favorable recovery patterns
Delayed staged reconstruction (“fix followed by flap”)	Nishida et al. [20]	Definitive fixation followed by delayed free-flap coverage	Gustilo-Anderson IIIB fractures when immediate reconstruction was not feasible	Bone union achieved without reported flap failure or infection recurrence in the series	Authors proposed delayed flap coverage as a feasible alternative in selected logistical or clinical scenarios
Delayed coverage beyond early reconstructive windows	Zamorano et al. [33]; Higgin et al. [34]	Soft-tissue coverage performed after prolonged intervals	Open tibial fractures and delayed reconstruction settings	Higher fracture-related infection rates and prolonged reconstructive burden reported	Delayed coverage was associated with worse infectious outcomes in selected cohorts
Timing in fracture-related infection reconstruction	Müller et al. [11]; Jagosz et al. [12]; Nasser et al. [19]	Timing adapted according to infection control, repeated debridement, and staged reconstruction	Fracture-related infection and post-traumatic osteomyelitis	Limb salvage remained feasible despite prolonged reconstruction and multiple procedures	Authors emphasized individualized sequencing and integration of debridement, fixation, and soft-tissue reconstruction

Historical Evolution and Conceptual Basis of the Orthoplastic Approach

The orthoplastic approach represents the evolution of extremity trauma care from a sequential, specialty-centered model towards an integrated reconstructive strategy in which skeletal stability, soft-tissue viability, vascular status, infection control, and functional recovery are addressed as interdependent components of the same clinical problem [5,6]. Its conceptual development reflects a gradual recognition that limb salvage cannot be achieved reliably by treating osseous injury and soft-tissue loss as separate entities. In complex extremity trauma, exposed bone, tendon, vascular structures, contamination, and devitalized tissue create a biological environment in which fixation without durable coverage may fail, while soft-tissue reconstruction without skeletal stability remains mechanically and functionally insufficient [6,7]. This interdependence provides the biological and functional rationale for orthoplastic care, supporting its role as a necessary model for the management of complex extremity trauma rather than merely an innovative surgical approach.

Historically, the principles underlying orthoplastic reconstruction were shaped by the management of severe open fractures and combat-related injuries, where delayed or fragmented treatment was associated with infection, nonunion, amputation, and poor functional recovery [5,8]. The early reconstructive literature established that timely debridement, stable fixation, and vascularized soft-tissue coverage were not isolated technical steps but sequential elements of a unified limb-salvage strategy [6,7]. The “fix and flap” paradigm further consolidated this concept by emphasizing coordinated skeletal stabilization and definitive soft-tissue coverage within an early reconstructive window, particularly in Gustilo-Anderson IIIB and IIIC injuries [5,6]. Contemporary orthoplastic practice extends this principle beyond timing alone, reframing reconstruction as a shared decision-making process between orthopedic trauma and plastic reconstructive teams.

Within this framework, the orthoplastic approach is not merely the simultaneous presence of orthopedic trauma and reconstructive plastic surgery teams during an episode of care. Rather, its defining characteristic is coordinated planning from the earliest stages of management, including joint assessment of limb viability, debridement strategy, fixation method, reconstructive options, infection risk, and anticipated functional outcomes [5,9]. This distinction is clinically relevant because a late plastic surgery consultation after definitive orthopedic fixation does not constitute true orthoplastic care. Rather, an orthoplastic model requires that reconstructive requirements influence orthopedic decisions from the outset, and that skeletal stabilization be planned in a way that facilitates timely vascularized coverage and rehabilitation [5,9]. Accordingly, orthoplastic surgery is best conceptualized as a time-sensitive, biologically integrated, and function-oriented model of extremity trauma care.

Clinical Burden of Complex Extremity Trauma and Reconstructive Need

Complex extremity trauma involves severe injuries that affect multiple anatomical and functional tissues simultaneously, requiring comprehensive reconstruction rather than isolated bone fixation. These cases often combine osseous disruption, soft-tissue loss, tendon exposure, vascular injury, contamination, and, in selected cases, neurological compromise. Because these structures differ in vascularity, healing potential, and tolerance to ischemia or infection, the clinical course is frequently complicated by wound breakdown, nonunion, fracture-related infection, flap failure, amputation risk, and prolonged functional impairment [6-8,10]. In this setting, the reconstructive problem begins at the time of injury, not after fracture stabilization has been completed.

The burden is particularly evident in severe open fractures, post-traumatic bone defects, and high-energy lower-extremity injuries, where the objective of care extends beyond limb preservation toward restoration of durable function. Contemporary reports emphasize that successful salvage requires more than anatomical reconstruction; it also depends on timely debridement, stable skeletal support, adequate vascularized coverage, infection control, and rehabilitation planning [7,11,12]. Long-term outcome studies further suggest that even when union and limb preservation are achieved, residual pain, impaired mobility, reduced quality of life, psychological distress, and delayed reintegration may persist, highlighting the need to evaluate recovery through functional, psychosocial, and patient-centered outcomes rather than surgical endpoints alone [13,14].

These features justify an early, coordinated orthoplastic approach involving orthopedic trauma and plastic reconstructive teams. Integrated planning allows bone stability, vascular status, soft-tissue reconstruction, and future rehabilitation to be considered simultaneously, reducing the risk that one therapeutic decision compromises another [6,15]. In specialized or protocol-driven settings, coordinated orthoplastic pathways have been associated with high limb-salvage rates and lower complication profiles, although outcomes remain influenced by injury severity, timing, institutional expertise, and resource availability [15,16,17]. Conversely, delayed or fragmented care increases operative burden, prolongs recovery, and may convert a potentially salvageable extremity into one with chronic infection, repeated reconstruction, or limited function. Therefore, complex extremity trauma should be approached from the outset as a reconstructive and functional emergency, not merely as an orthopedic injury requiring later soft-tissue closure.

Core Principles of Orthoplastic Management

The core principles of orthoplastic management are contamination control, skeletal stability, vascularized soft-tissue coverage, preservation of perfusion, and functional rehabilitation. In complex extremity trauma, these principles are not independent therapeutic steps, but mutually dependent elements of a single reconstructive pathway. Thorough debridement remains the first decisive intervention because residual devitalized tissue, bacterial contamination, and poorly vascularized wound beds increase the risk of fracture-related infection, nonunion, flap compromise, and eventual limb loss [18,19]. When necessary, repeated debridement allows progressive clarification of tissue viability and prepares the wound for definitive fixation and coverage.

Skeletal stabilization must be selected according to fracture pattern, contamination burden, bone loss, and the condition of the surrounding soft-tissue envelope. Internal fixation may be appropriate after adequate debridement and antimicrobial coverage, whereas staged fixation strategies may be necessary in contaminated wounds, unstable patients, or defects requiring delayed reconstructive planning [20,21]. The essential orthoplastic principle is that fixation should not be chosen in isolation: implants, external frames, bone reconstruction strategies, and flap options must be planned together to avoid compromising vascular access, soft-tissue coverage, or future rehabilitation [22,23]. In this sense, stabilization is not merely mechanical; it is a reconstructive decision.

Definitive soft-tissue coverage with well-vascularized tissue is another central element of orthoplastic care. Vascularized coverage protects exposed bone, tendons, vessels, and implants, obliterates dead space, improves local perfusion, and creates a more favorable environment for infection control and bone healing [21,24]. Contemporary experience with free flaps, combined flaps, pedicled flaps, and vascularized bone-containing constructs demonstrates that reconstructive choice should be tailored to the defect, patient risk profile, and functional goal rather than dictated by technical preference alone [24-26]. Preserving blood supply through atraumatic tissue handling and early repair of vascular injury remains fundamental, particularly when limb viability depends on timely revascularization before definitive reconstruction.

The defining feature of orthoplastic management is therefore integrated decision-making. When orthopedic and plastic reconstructive teams plan jointly from the beginning, the sequence of debridement, fixation, antimicrobial strategy, vascular repair, soft-tissue coverage, and rehabilitation becomes coordinated rather than reactive [18,22]. This approach also allows contingency planning in complex scenarios, including infected fields, bone defects, flap failure, and staged reconstruction [19,27]. Ultimately, orthoplastic care is not defined by the use of a specific flap or fixation method, but by the capacity to align surgical priorities around the biological requirements of healing and the functional needs of the patient.

Timing and Sequencing in Reconstruction

Timing is one of the defining variables of orthoplastic reconstruction because contamination control, skeletal stabilization, vascular repair, and soft-tissue coverage are biologically interdependent. In the reviewed literature, reconstruction is commonly framed as immediate, early, or delayed; however, contemporary orthoplastic practice increasingly interprets timing not as an isolated chronological threshold, but as the coordination of sequential decisions within the earliest safe reconstructive window. Early debridement remains essential to remove devitalized tissue and reduce bacterial burden, but the quality of debridement, senior surgical involvement, antibiotic timing, and integration with definitive reconstruction are now considered more clinically relevant than rigid adherence to historical time rules [28]. In this context, timing becomes a marker of system performance as much as of surgical decision-making.

The classic principle of early definitive coverage, particularly within the first 72 hours, remains a central reference point in severe open fractures. Institutional protocols and orthoplastic pathways have shown that coordinated early coverage can reduce infection, nonunion, reoperation burden, and delays in rehabilitation when compared with fragmented or delayed models [15,29,30]. Large contemporary series of Gustilo-Anderson IIIB/C tibial fractures support this concept, showing that coordinated orthoplastic management with early coverage is associated with lower deep infection rates, reduced nonunion, fewer procedures, and improved functional recovery [15]. Similarly, audits of BOAST4-guided care suggest that adherence to structured timing targets improves consistency in decision-making, although real-world compliance remains limited by operating room availability, microsurgical resources, patient physiology, and institutional logistics [31,32]. 

Nevertheless, the literature also indicates that timing should be clinically adaptive rather than dogmatic [30,31]. In selected cases, staged reconstruction may be appropriate when contamination, soft-tissue viability, vascular status, or patient instability preclude safe immediate coverage. The “fix followed by flap” strategy illustrates that delayed free flap coverage after definitive internal fixation can be feasible when performed within a controlled protocol, although the small size and retrospective nature of such evidence limit broad generalization [20]. Other studies suggest that coverage before later biological thresholds, such as within 7-12 days, may still reduce fracture-related infection when early reconstruction is not feasible, but outcomes become increasingly dependent on repeated debridement, wound control, and coordinated planning [33,34]. Therefore, the strongest message from the literature is not that every patient must undergo reconstruction at an identical time point, but that delays should be intentional, protocol-driven, and biologically justified rather than the result of fragmented care.

Skeletal Stabilization and Soft-Tissue Coverage as Integrated Strategies

Skeletal stabilization and soft-tissue coverage should be understood as complementary components of a single reconstructive strategy rather than as independent surgical events. In complex extremity trauma, stable fixation restores alignment, reduces pathological motion, and provides the mechanical environment required for bone healing; however, fixation alone cannot compensate for devitalized or insufficient soft tissue. Conversely, vascularized coverage protects exposed bone, tendons, vessels, and implants, restores local perfusion, obliterates dead space, and contributes to infection control. For this reason, orthoplastic decision-making requires that the fixation method, timing of coverage, anticipated flap requirements, and rehabilitation goals be planned together from the outset [12,24,35].

This principle becomes especially relevant in defects involving bone loss, exposed implants, or fracture-related infection. In these scenarios, achieving both union and infection control requires coordinated surgical debridement, dead-space management, skeletal reconstruction, and vascularized soft-tissue coverage. Retrospective orthoplastic series of lower-leg fracture-related infection suggest that successful reconstruction depends on combining radical debridement with stable fixation and soft-tissue reconstruction, rather than relying on any single intervention in isolation [35]. Similarly, experience with post-traumatic osteomyelitis emphasizes that limb preservation requires an algorithmic strategy integrating infection eradication, skeletal support, and durable coverage, although the available evidence remains limited by small samples and retrospective designs [12]. These findings support the concept that vascularized tissue is not merely a closure method but a biologically active component of reconstruction.

Contemporary reconstructive strategies further illustrate this integration. Combined free flaps, vascularized bone-containing flaps, and techniques such as Masquelet-assisted reconstruction have been used to address simultaneous bone and soft-tissue deficits, particularly when conventional fixation or coverage alone would be insufficient [22,24]. Series using chimeric or osteocutaneous flaps show that tailored reconstruction may restore both structural support and soft-tissue integrity while maintaining the possibility of functional rehabilitation [22,26,36]. However, these studies are generally small and technically heterogeneous, limiting broad comparisons. Their value lies less in proving the superiority of one flap or fixation construct and more in demonstrating that complex trauma reconstruction must be designed around the combined biological requirements of bone, soft tissue, perfusion, and function.

In cases with vascular, tendinous, or neural compromise, sequencing becomes particularly critical. Restoration of perfusion, stabilization of the skeletal framework, and coverage of exposed functional structures must be prioritized according to limb viability and reconstructive feasibility. The orthoplastic model therefore does not prescribe a single universal sequence; instead, it requires coordinated judgment to determine the appropriate sequence: whether immediate fixation and coverage are feasible, whether staged reconstruction is safer, or whether adjunctive strategies such as local antibiotic delivery or repeat microsurgical reconstruction are warranted [19,21,27]. Ultimately, the integration of skeletal stabilization and soft-tissue coverage is the clinical mechanism through which orthoplastic care transforms limb salvage from a technical objective into a functional reconstructive pathway.

Organization of Care and Interdisciplinary Models

The orthoplastic approach depends not only on surgical expertise but also on the organization of care. A truly orthoplastic system is defined by early joint assessment, coordinated operative planning, shared responsibility for sequencing, and access to both orthopedic trauma and reconstructive plastic surgery capabilities. This differs from a traditional referral model in which plastic surgery is consulted only after fixation has been completed or after soft-tissue complications have emerged. In complex extremity trauma, the timing of debridement, stabilization, vascular assessment, soft-tissue coverage, and rehabilitation is highly dependent on institutional coordination; therefore, outcomes are reshaped by the care pathway as much as by the individual procedure [9,15,31].

Evidence from protocol-driven centers suggests that institutional organization can reduce variability and improve clinical performance. Implementation of an orthoplastic treatment protocol in a tertiary trauma unit was associated with lower complication rates, supporting the value of standardized pathways for open tibial fractures [9]. Similarly, experience from major trauma centers evaluating adherence to BOAST4 guidelines shows that even when ideal timing targets are difficult to achieve consistently, structured multidisciplinary assessment and defined referral pathways may help maintain low deep infection rates and improve the predictability of care [31]. Large institutional series of Gustilo-Anderson IIIB/C tibial fractures further support the association between formal orthoplastic collaboration, early coverage, fewer procedures, and improved limb salvage outcomes [15]. These findings suggest that orthoplastic care functions best when it is embedded within a system rather than dependent on ad hoc consultation.

Institutional volume and reconstructive capacity also influence outcomes [21]. Higher-volume centers with established orthoplastic collaboration appear better positioned to provide microsurgical coverage, manage flap complications, coordinate staged reconstruction, and offer timely escalation when initial strategies fail [10,19]. Multicenter audits of open tibial fracture management reinforce this point by showing that guideline implementation varies across major trauma centers, reflecting differences in infrastructure, staffing, theatre access, and local pathways [37]. Qualitative evidence further indicates that barriers to implementation are not limited to knowledge of guidelines but include workflow fragmentation, cultural resistance, resource constraints, and poor translation of written protocols into daily clinical practice [37].

Importantly, the orthoplastic model can be adapted beyond ideal tertiary-center environments, but its effectiveness depends on preserving its core organizational principles. Humanitarian and resource-limited experiences demonstrate that interdisciplinary limb-salvage models may be implemented under constrained conditions, although infection, nonunion, and follow-up limitations remain major challenges [38,39]. In the reviewed literature, resource-limited settings encompassed humanitarian missions, conflict-affected regions, and healthcare systems with limited access to reconstructive infrastructure, microsurgical capabilities, specialized personnel, and multidisciplinary trauma services (Table 2). These settings highlight that orthoplastic care should be viewed as a systems-based model: when microsurgical resources are limited, the essential objective is still early coordination, disciplined debridement, rational stabilization, timely coverage when feasible, and rehabilitation-oriented planning. Thus, the organizational lesson is clear: orthoplastic care is not merely the coexistence of two specialties but the creation of a coordinated trauma pathway capable of converting surgical expertise into reproducible patient outcomes.

Clinical Outcomes and Evidence Synthesis

The available evidence suggests that orthoplastic management is associated with favorable clinical outcomes in complex extremity trauma, particularly when care is delivered through coordinated protocols rather than fragmented referral pathways. Across the included studies, the most consistently reported outcomes were infection, amputation, limb salvage, reoperation burden, time to definitive coverage, length of hospital stay, and functional recovery. Although heterogeneity in study design, injury severity, follow-up duration, and institutional resources limits direct comparison, overall, the available evidence suggests that early multidisciplinary coordination is associated with improved limb preservation and fewer complications [9,15,31,35].

Infection remains one of the most important outcome measures in severe open fractures and post-traumatic reconstruction. Protocol-driven orthoplastic care was associated with low reported infection rates in several cohorts, including 1.6% after implementation of an orthoplastic protocol in a Finnish tertiary trauma unit and 4.5% deep infection in a UK major trauma center audit despite limited adherence to the 72-hour coverage target [9,15]. In contrast, studies conducted in more complex or resource-constrained settings reported substantially higher infection rates, such as 44.5% in a humanitarian cohort from Gaza and 36.6% fracture-related infection in a selected cohort of young patients with Gustilo-Anderson IIIB tibial fractures treated with later soft-tissue coverage [38,39]. These differences likely reflect not only surgical timing but also injury burden, contamination, system capacity, access to reconstructive expertise, and follow-up heterogeneity.

Limb salvage and amputation outcomes also favor coordinated reconstruction, although interpretation must remain cautious because many studies lack randomized comparators. Large and protocol-based series reported high limb-salvage rates, including 96.6% in a 746-patient Gustilo-Anderson IIIB/C cohort from China, 98.4% after implementation of an institutional orthoplastic protocol, and 96.2% in a UK major trauma center audit [9,15,31]. Smaller series also reported high salvage rates, including 94.4% after microsurgical management of free flap compromise or failure and 95.1% in lower-leg fracture-related infection managed with orthoplastic reconstruction [27,36]. However, these results should not be interpreted as proof of universal superiority because case selection, center experience, and availability of salvage resources strongly influence outcomes.

Beyond limb preservation, the reviewed evidence increasingly emphasizes reconstructive efficiency and functional recovery. Multidisciplinary pathways may reduce the number of procedures, shorten time to definitive coverage, and limit prolonged inpatient care when compared with delayed or fragmented models. For example, formal orthoplastic protocols reported definitive coverage within short intervals and hospital stays of around 17.5 days in one UK audit, whereas fracture-related infection cohorts reported longer admissions, reflecting the burden of delayed or complicated reconstruction [16,26,35]. Functional reporting remains inconsistent, but selected studies used instruments such as the Lower Extremity Functional Scale, Johner-Wruhs criteria, modified Enneking score, AOFAS, and SF-12, suggesting a gradual shift from purely surgical endpoints toward patient-centered recovery [16,30,33]. Overall, the evidence supports the orthoplastic approach as a clinically coherent model associated with lower complication profiles and high limb-salvage rates in experienced settings, while also underscoring the need for standardized functional outcomes, prospective comparisons, and better adjustment for injury severity.

Barriers to Implementation, Evidence Gaps, and Future Directions

A major barrier to interpreting the orthoplastic literature is the absence of a uniform operational definition of the “orthoplastic approach.” Across the reviewed studies, the term is used to describe a wide spectrum of care models, ranging from staged referral between orthopedic and plastic surgery teams to formal institutional protocols, co-managed trauma pathways, and high-volume reconstructive centers. This conceptual variability limits direct comparison across studies because the same label may refer to substantially different levels of integration, timing, team structure, and reconstructive capacity [6,7,29]. As a result, the literature often supports the general value of interdisciplinary reconstruction but provides limited detail on how such models are organized, measured, or reproduced across institutions.

Methodological limitations further constrain the robustness of the available conclusions. Much of the evidence consists of retrospective cohorts, small institutional series, case reports, technical reports, and narrative reviews, often without concurrent control groups or standardized adjustment for injury severity [16,35,40,41]. Several included studies provide valuable technical or contextual insights but have limited external validity because of small sample size, highly selected populations, heterogeneous indications, or absence of comparative analysis [35,40,41]. Even larger cohorts remain vulnerable to confounding by center experience, referral patterns, resource availability, and differences in timing definitions. Therefore, although the direction of evidence favors coordinated orthoplastic care, the current literature remains better suited to hypothesis generation and clinical synthesis than to definitive causal inference.

Outcome reporting is another important gap. Infection, amputation, flap failure, union, and limb salvage are frequently reported, but long-term functional recovery, return to work, pain, quality of life, and patient-reported outcomes remain inconsistently captured. Some studies incorporate validated measures such as LEFS, AOFAS, SF-12, Johner-Wruhs criteria, or modified Enneking/MSTS scores, but these instruments are not used uniformly, and follow-up duration varies considerably [14,26,42]. The QUINTET cohort illustrates the importance of shifting beyond limb salvage toward quality-of-life assessment, yet such prospective, patient-centered approaches remain uncommon in the orthoplastic trauma literature [13]. This limits the ability to determine whether a technically salvaged limb translates into durable functional recovery.

Future research should prioritize prospective multicenter designs, standardized definitions of orthoplastic care, and core outcome sets that include both surgical and patient-centered endpoints. Studies should report not only procedures and complications but also organizational variables such as timing of plastic surgery involvement, multidisciplinary team composition, availability of microsurgical coverage, institutional volume, escalation pathways, rehabilitation access, and adherence to open-fracture protocols [36,39]. Qualitative and implementation-science research may also be necessary to explain why written guidelines fail to translate into consistent practice across trauma systems [40-42]. Future studies should also evaluate the cost-effectiveness and sustainability of orthoplastic pathways, particularly in healthcare systems with variable access to microsurgical infrastructure, multidisciplinary teams, and rehabilitation services. Without such methodological and organizational standardization, the orthoplastic approach will remain a clinically compelling model supported by convergent observational evidence but not yet fully characterized as a reproducible standard of care across diverse healthcare environments.

Discussion

The findings of this narrative review support the orthoplastic approach as a clinically coherent model for the management of complex extremity trauma. Rather than representing a simple collaboration between orthopedic and plastic surgery, orthoplastic care reframes limb reconstruction as an integrated, time-sensitive process in which skeletal stability, soft-tissue viability, vascular status, infection control, and functional recovery are addressed simultaneously [5-7]. This distinction is clinically relevant because fragmented or sequential care may delay definitive coverage, prolong exposure of critical structures, and increase the risk of infection, nonunion, reoperation, and functional impairment [6,7,15].

Early coordination emerged as one of the most consistent determinants of outcome, with reconstructive timing functioning as a marker of multidisciplinary system performance rather than as a rigid chronological threshold. Early coordinated reconstruction, particularly within established “fix-and-flap” or institutional orthoplastic pathways, was associated with lower infection rates, fewer complications, and high limb-salvage rates in several cohorts [9,15,20,31]. However, the evidence also suggests that timing should not be interpreted as a rigid rule. In selected patients with contamination, physiological instability, or complex reconstructive requirements, staged strategies may be appropriate when delays are intentional, protocol-driven, and supported by repeated debridement, infection control, and planned vascularized coverage [11,12,19,20].

A central implication of the reviewed literature is that bone and soft tissue cannot be managed as independent reconstructive problems. Stable fixation provides the mechanical environment required for healing, but its success depends on a viable, vascularized soft-tissue envelope. Conversely, flap coverage without adequate debridement, infection control, and skeletal stability is unlikely to produce durable functional recovery [11,18,19,24]. This biological interdependence explains why orthoplastic care appears most effective when debridement, fixation, antimicrobial strategy, soft-tissue coverage, and rehabilitation are planned as a unified sequence rather than as isolated procedures.

The organizational dimension of orthoplastic care is equally critical. Protocol-driven centers, multidisciplinary pathways, and early joint decision-making were repeatedly associated with improved consistency of care and favorable clinical outcomes [9,15,31]. In contrast, delayed consultation models and resource-variable settings highlight the vulnerability of patients when reconstructive planning depends on availability rather than predefined systems [37-39]. These findings suggest that orthoplastic success is not determined solely by technical expertise but by the capacity of a trauma system to deliver coordinated reconstruction within the optimal therapeutic window.

Despite encouraging trends, the current evidence remains limited. Most available studies are retrospective, single-center, and heterogeneous in definitions, injury severity, timing thresholds, and outcome reporting. Functional and patient-centered outcomes remain underreported, although studies using LEFS, SF-12, AOFAS, or other instruments reinforce the need to move beyond limb salvage as the only measure of success [13,14,27,30]. Future research should prioritize prospective multicenter designs, standardized definitions of orthoplastic care, and core outcome sets that integrate infection, union, limb salvage, function, quality of life, return to work, and organizational variables.

Overall, the orthoplastic approach represents a paradigm shift in complex extremity trauma: from specialty-based intervention to integrated reconstructive care; from delayed reaction to time-sensitive planning; and from limb preservation alone to functional restoration. Its greatest contribution is not the use of a specific flap, fixation method, or protocol but the creation of a coordinated model in which physiological, surgical, and organizational decisions are aligned toward durable recovery. Importantly, the applicability of this model in resource-limited settings depends on adapting its core principles to local reconstructive capacity, multidisciplinary availability, and rehabilitation infrastructure.

## Conclusions

The orthoplastic approach should be understood as a comprehensive reconstructive framework for complex extremity trauma, in which skeletal stabilization, soft-tissue coverage, vascular integrity, infection control, and functional recovery are addressed as interdependent priorities. The available evidence supports early multidisciplinary coordination as a key determinant of more coherent surgical sequencing, improved reconstructive efficiency, and clinically meaningful limb-salvage strategies. Although outcomes remain influenced by injury severity, timing, institutional resources, and heterogeneity in the literature, orthoplastic care provides a pragmatic model for aligning biological principles with functional goals. Ultimately, this approach represents a paradigm shift from fragmented, specialty-based intervention toward coordinated trauma reconstruction aimed at durable limb salvage, biological healing, and meaningful functional recovery. Future implementation should also consider the sustainability and cost-effectiveness of orthoplastic pathways across diverse healthcare environments.
